# Strong effects of food quality on host life history do not scale to impact parasitoid efficacy or life history

**DOI:** 10.1038/s41598-023-30441-1

**Published:** 2023-03-02

**Authors:** Leslie A. Holmes, William A. Nelson, Stephen C. Lougheed

**Affiliations:** 1 University of Lethbridge, 4401 University Drive West, Lethbridge, AB T1K 3M4 Canada; 2 Queen’s University, 116 Barrie Street, Kingston, ON K7L 3N6 Canada

**Keywords:** Community ecology, Population dynamics, Ecology

## Abstract

Parasitoids are small insects, (e.g., small wasps or flies) that reproduce by laying eggs on or within host arthropods. Parasitoids make up a large proportion of the world’s biodiversity and are popular agents of biological control. Idiobiont parasitoids paralyze their hosts upon attack and thus are expected to only target hosts large enough to support offspring development. Host resources generally impact host attributes and life histories including size, development, and life span. Some argue slow host development in response to resource quality increases parasitoid efficacy (i.e., a parasitoid’s ability to successfully reproduce on or within a host) due to longer host exposure to parasitoids. However, this hypothesis is not always supported and does not consider variation in other host traits in response to resources that may be important for parasitoids (e.g., variation in host size is known to impact parasitoid efficacy). In this study we test whether trait variation within host developmental stages in response to host resources is more important for parasitoid efficacy and life histories than trait variation across host developmental stages. We exposed seed beetle hosts raised on a food quality gradient to mated female parasitoids and measured the number of hosts parasitized and parasitoid life history traits at the scale of host stage- and age-structure. Our results suggest host food quality does not cascade to impact idiobiont parasitoid life histories despite large food quality effects on host life history. Instead, variation in host life histories across host developmental stages better predicts parasitoid efficacy and life histories, suggesting finding a host in a specific instar is more important for idiobiont parasitoids than finding hosts on or within higher quality resources.

## Introduction

Parasitoids are ubiquitous in terrestrial arthropod communities^1^. Parasitoid life history strategy resembles that of parasites and predators. Like parasites, immature parasitoids survive by feeding on the bodies of animals and require only a single arthropod body (herein referred to as host) to complete their development; however, unlike parasites, but like predators, parasitoids kill their hosts^2^. Comprising more than 50% of all described animal species, parasitoids and their herbivore hosts make up a large proportion of the world’s biodiversity^3,4^, and affect ecosystem function and community structure^5,6^. For example, in the absence of parasitoids, when raised on fava beans, pea aphids competitively exclude their resource competitor, the vetch aphid^7^. However, in the presence of a shared parasitoid, stable populations of all three invertebrate species persist with little to no fluctuation in species abundance^7^. Moreover, parasitoids may be more important for controlling invertebrate herbivore populations than ground dwelling invertebrate predators^8^, and for more than a century, they have been employed as agents of biological control, comprising a multi-billion-dollar global industry^9^.

A key factor influencing parasitoid fitness is host susceptibility^2,10^ (i.e., how likely a host will be successfully parasitized when encountered by a parasitoid), and how susceptibility changes throughout host ontogeny^11,12^. Host susceptibility is determined by host life history including growth patterns, defense mechanisms, and life span^2^. Some parasitoids are specialists, parasitizing single host species, while others are generalists, parasitizing several host species with similar life histories^13^. For both specialists and generalists, many parasitoids target a specific stage or size of host that provide enough resources to support the growth and development of their offspring^13,14^. This is particularly important for parasitoids that paralyze their hosts (i.e., idiobiont parasitoids that parasitize larval hosts) because paralysis prevents any further host growth and development. Thus, idiobiont parasitoid success largely depends on host size at the time of attack. Moreover, host size is positively correlated with parasitoid survivorship, body size^15,16^, and fecundity^17,18^ suggesting that idiobiont parasitoids should attack larger hosts. Consequently, idiobiont parasitoid hosts are typically only susceptible to their parasitoids at specific sizes or later stages of juvenile ontogeny^2^. Thus, environmental factors that alter host growth and development in these latter stages of host ontogeny have the potential to scale-up to impact parasitism rates and parasitoid fitness.

Food quality and its availability are two environmental factors that impact growth and development of host insects, where poor food quality often results in longer development times^19,20^ and smaller body sizes at maturity^21^. The ability to experimentally manipulate host life histories in response to food has prompted investigations into life history variation effects on host susceptibility to parasitism (reviewed in Williams^22^; see also a meta-analysis by Chen and Chen^23^). However, these studies typically group all instar stages into a single ‘juvenile stage’, assuming changes in host vital rates are constant throughout juvenile ontogeny despite knowing that food consumption and digestibility can influence insect ontogeny^24,25^, and that parasitoids typically target specific stages of development and/or sizes.

In a previous study, we demonstrated that food quality effects on seed beetle host growth and development are not consistent across host developmental stages^26^. Specifically, we showed that late instar hosts consuming low food quality resources have longer development times and reduced growth than hosts consuming higher food quality resources, but early instar hosts showed small differences in growth and development among food quality treatments (90%, 95%, and 100% black-eye pea artificial seeds)^26^. The distinction between these life history traits is important because increased length of host development, all else being equal, would likely benefit parasitoids by extending the time hosts are in vulnerable stages, whereas decreased growth would disadvantage parasitoids. Because host size is an important predictor of parasitoid success and fitness^17,18^, it is not clear whether smaller hosts that develop slowly, and thus are exposed to parasitoids for longer periods, are as susceptible to parasitoids as larger hosts that develop more quickly with shorter exposure times.

The direct dependence of parasitoids on their insect hosts, and of insect hosts on their plant resources, creates the potential for an indirect link between plant resources and host-parasitoid interactions. Resources can have large cascading effects on higher trophic consumers (reviewed in^27,28^), and higher trophic consumers (i.e., predators and parasitoids) can have similar if not larger cascading effects on lower trophic levels^28^. However, studies showing either the presence or absence of bottom-up trophic cascades in resource-host-parasitoid communities are approximately equal in number^22,23,27–30^ and thus whether these cascades do indeed occur is uncertain. Characterizing resource effects on host-parasitoid communities will provide important life history data to predict resource-host-parasitoid relationships and dynamics and help garner a better understanding of when resources are expected to cascade, and when they are not.

Here we study parasitoid efficacy and life history when parasitizing hosts that have fed on a food quality gradient. We use the seed beetle host *Callosobruchus maculatus* (Fabricius) (Coleoptera: Bruchidae) because it is a concealed herbaceous host of legume seeds, easily maintained in the lab on natural and artificial seeds and is parasitized both in the lab and in the field by its idiobiont parasitoid, *Anisopteromalus calandrae* (Howard) (Hymenoptera: Pteromalidae). We exposed independent samples of 5 to 60-day-old hosts raised on artificial seeds varying in food quality to mated female parasitoids for 24 h and quantified: number of hosts parasitized, host stage of development at the time of parasitism, and parasitoid development time, survivorship, emergence mass, sex, and hind tibia length. By experimentally manipulating host food quality, we were able to quantify parasitoid efficacy and life history across the entire span of host development (i.e., age-structure of the host). Further, by identifying stages of host development for each host age exposed to a parasitoid, we combined stage- and age-structure approaches to characterize the importance of host variation within and among stages of host development on parasitoid efficacy and life history. From our previous study^26^ and literature supporting bottom-up cascades of host resources in resource-host-parasitoid food webs^27,28^, we expect to find host resources indirectly impacting idiobiont parasitoid efficacy and life history. Specifically, we expect hosts consuming lower seed qualities would be more susceptible to their idiobiont parasitoids due to longer exposure to parasitoids^22,23^, and produce smaller parasitoids that are allocated to males^31^.

## Materials and methods

### *Callosobruchus maculatus* life history

Stock populations of hosts, *C. maculatus,* were reared on black-eye peas *Vigna unguiculata* and maintained in a Conviron CMP 3244 climate-controlled growth chamber at 28 °C, 75% relative humidity, and a 12:12 [L:D] fluorescent light regime. Female *C. maculatus* lay their eggs on surfaces of seeds. First instar larvae hatch from their eggs and burrow into seeds, feeding on the inner flesh. Larvae develop through four instars, each time molting their exoskeleton and head capsule^32^. At the end of their fourth instar, larvae will molt a final time, and enter the pupal stage, where they metamorphose into adults. Adults emerge from seeds sexually mature. This developmental period from egg to adult takes approximately 28 days in the above laboratory conditions; however, on low artificial seed qualities, development can take up 60 days^26^.

### *Anisopteromalus calandrae* life history

Stock populations of parasitoids, *A. calandrae* were reared on 16–18-day old stock populations of naïve *C. maculatus* hosts (i.e., not previously exposed to parasitoids) and maintained in the same climate-controlled growth chamber as stock populations of *C. maculatus*. *Anisopteromalus calandrae* is a generalist idiobiont ectoparasitoid that typically attacks older instar larvae of various seed and grain beetle species. Upon attack, *A. calandrae* paralyze their hosts causing a cessation of development^33^. After paralysis, *A. calandrae* oviposit a single egg on the outside integument of host larvae. Hatched parasitoid larvae develop by feeding on host larvae, eventually killing their hosts. After metamorphosing into adults, parasitoids emerge from host seeds or grains sexually mature. This developmental period from egg to adult takes approximately 12–14 days in the above laboratory conditions. *Anisopteromalus calandrae* are haploid-diploid, meaning that females can choose to produce diploid female offspring by fertilizing their eggs with stored sperm from mating, or produce haploid male offspring (either because they have not mated or have chosen not to fertilize their eggs with stored sperm).

### Artificial black-eye peas

Confounding variation in plant physiology, plant defense compounds, and overall seed size can arise when using different species of natural seeds to create a food quality gradient. To control such variation, we created artificial seeds from black-eye pea flour. We created seeds varying in quality by adding different amounts of indigestible crude fiber and lignin (filler) from milled peanut hulls to black-eye pea flour in the following proportions: 90:10; 95:5; 100:0 of flour:filler as % dry mass^34^. Black-eye peas and raw peanuts used to create artificial seeds were commercially sourced, complying with all international, national, and institutional agency guidelines.

### Characterizing resource quality effects on parasitoid efficacy and life history

Following similar methods in a previous experiment^26^, *C. maculatus* eggs were collected from stock populations and transferred to artificial seeds (i.e., 90%, 95%, and 100% black eye pea artificial seeds), such that each artificial seed had a single egg and each egg used in our experiment had a unique mother. The technique of transferring seed beetle eggs to artificial seeds is described in Holmes et al*.*^34^. For each food quality treatment, 56 seed beetle eggs were transferred to 56 artificial seeds (i.e., one egg for each day of host development from 5-days-old to 60-days-old).

Beginning on day 5 of the experiment, (i.e., 5-day-old hosts), a single artificial seed was removed each day for 55 days (i.e., 60 days of host development from oviposition) from each seed quality treatment in each replicate and exposed to a single mated stock female *A. calandrae* for 24 h. After parasitoid exposure, host artificial seeds were monitored daily for 60 days for parasitoid or weevil emergence and the mating status of each female parasitoid was confirmed in a secondary assay (see supplementary materials). Upon adult emergence of either host (i.e., when a parasitoid larva did not consume the weevil larva) or parasitoid, samples were placed in a − 10 °C freezer until further processing; all other samples were placed in a − 10 °C freezer after 60 days of observation post parasitoid exposure. There were 54 replicates in each treatment, such that the total number of hosts exposed to parasitoids (54 replicates × 56 days × 3 treatments) was: 9072 (Supplementary Material Fig. S1).

Frozen artificial seeds were dissected using a scalpel under a dissecting microscope to retrieve host weevils that had not emerged. For each host that had not emerged from its artificial seed, we noted if it was parasitized by the presence of an egg, immature, or adult parasitoid, and identified the stage of host development by counting the number of molted host head capsules recovered during dissection. Because hosts are paralyzed by *A. calandrae* upon attack, the number of host head capsules recovered during dissection reflects host developmental stage at the time of parasitism.

Frozen adult parasitoids were left at room temperature for 10 min before any measurements were taken. We measured the left hind tibia length using 6× magnification and calibrated ocular micrometer for each adult parasitoid that emerged from a host, and quantified emergence mass for a random subset of adults (N = 371) using a microbalance scale (Mettler Toledo XP6, Canada). This allowed us to test for a correlation between parasitoid emergence mass and hind tibia length and use hind tibia length as a proxy for parasitoid size and fitness^35^. Emergence mass was measured three times and the average of these three measurements was used for all subsequent analyses.

### Testing for differences in parasitoid efficacy and life history

We used generalized linear models (GLMs) and generalized additive models (GAMs) to characterize parasitoid efficacy, emergence mass, hind tibia length, development time, and sex ratio across categorical covariates including, stages of host development at the time of host parasitism and a seed quality gradient of artificial seeds hosts consumed, and across the numerical covariate host age, respectively using the mgcv package^36,37^. Table 1 outlines the systematic component of the statistical models, sample sizes, and error distributions for each analysis. For each GAM, we modeled non-linear relationships between response variables and host age at the time of parasitism using smooth functions by fitting a thin plate regression spline^38^ to covariate levels (e.g., seed quality levels). All statistical analyses were done using the R software environment version 3.6.2^39^.

**Table 1:** Statistical methods for characterizing parasitoid efficacy and life history on hosts consuming a gradient of artificial seed quality. Model selection with Akaike Information Criteria (AIC) and quasi-AIC was used to evaluate statistical significance. Stage-structure analyses compared suites of generalized linear models (GLMs), while age-structure analyses compared suites of generalized additive models (GAMs). Response (Y) and predictor variables are indicated in the first and third columns, respectively. For all analyses, except where noted, Q is the artificial seed quality hosts consumed (90%, 95% and 100% black-eye pea flour), S is the stage of host development (1st instar, 2nd instar, 3rd instar, 4th instar, pupal and adult) at the time of parasitoid attack, T is the log hind tibia length (mm) of parasitoids that emerged from parasitized hosts, s() is the smoothing function of A, where A is the age of hosts at the time of parasitoid attack. The by = Q and by = G options allow for GAM evaluations of whether each food quality level (Q) or parasitoid sex (G) has a different smooth function by host age.

Analyses response variable (Y)	Sample size	Systematic	Error distribution	AIC selected top model
Parasitoid efficacy				
Stage-structure of host				
Proportion of hosts parasitized	6140	Y ~ Q + S	Quasi Binomial with a logit link	Y ~ Q + S
Proportion of parasitized hosts producing adult parasitoids	1410	Y ~ Q + S	Binomial with a logit link	Y ~ S
Age-structure of host				
Proportion of hosts parasitized	6140	Y ~ Q + s(A, by = Q)	Binomial with a logit link	Y ~ Q + s(A, by = Q)
Parasitoid life history				
Stage-structure of host				
Parasitoid emergence mass (mg)	375	Y ~ T + G	Gaussian with an identity link	Y ~ T * G
Parasitoid hind tibia length (mm)	1131	Y ~ Q * S * G^‡^	Gaussian with an identity link	Y ~ G * S^‡^
Parasitoid development time (days)	1197	Y ~ Q + S + G^‡^	Gamma with an inverse link	Y ~ G
Proportion of female parasitoids	1158	Y ~ Q + S^‡^	Binomial with a logit link	Y ~ S + Q^‡^
Age-structure of host				
Parasitoid development time (days)	1197	Y ~ Q + G + s(A, by = Q) + s(A, by = G)	Gamma with an inverse link	Y ~ G + s(A)
Proportion of female parasitoids	1158	Y ~ Q + s(A, by = Q)	Binomial with a logit link	Y ~ Q + s(A)
Parasitoid Log hind tibia length (mm)	1131	Y ~ Q + G + s(A, by = Q) + s(A, by = G)	Gaussian with an identity link	Y ~ Q + G + s(A, by = Q) + s(A, by = G)
Host log dry biomass (mg)	2835	Y ~ Q + s(A, by = Q)^‡‡^	Gamma with an inverse link	Y ~ Q + s(A, by = Q)^‡‡^

^‡^Where S is the stage of hosts (2nd instar, 3rd instar, 4th instar, pupal and adult) at the time of parasitoid attack.

^‡‡^Where A is the age of hosts.

Statistical significance was evaluated using model selection^37,40,41^ with Akaike Information Criteria (AIC). Quasi Akaike Information Criteria (qAIC) was used where appropriate; their values calculated using the bbmle package^42^. The general linear hypothesis testing function glht in the Multcomp package^43^ was used for post-hoc analyses of GLMs, where applicable, and pairwise comparisons of the estimated smooth functions of GAMs were computed following Rose et al*.*^44^; p values were adjusted for Type I error using the Bonferroni method.

While our methods can identify developmental stages of parasitized hosts, because parasitoids consume host fat bodies, we could not characterize host size at the time of parasitism directly. To get a sense of how host size might impact parasitoids, we used data^45^ from a previous experiment not reported in Holmes et al*.*^26^ that used the same experimental methods to characterize the age distribution of host size across the same gradient of seed quality used in this study. With an age distribution of host size and an age distribution of parasitoid size (e.g., hind tibia length) across the same seed quality gradient, we can estimate host size at the time of parasitoid attack across each seed quality treatment and infer its role in parasitoid efficacy and life history. Because we found that no adult parasitoids emerged from parasitized first instar hosts in the current study, for comparison we excluded first instar hosts from our age distribution of host size analysis. Statistical analysis followed the same steps as above for generalized additive model analysis.

To test whether the trait variation within host stages in response to host resources is more important for parasitoid efficacy than the trait variation across host stages, we estimated the daily risk (hazard rate) of hosts being parasitized for each seed quality treatment within each stage of host development. To do this, hazard rate models require knowledge of the stage of host development at the time of an event of interest (i.e., the time hosts are exposed to their parasitoids with an outcome of either successful or unsuccessful parasitism). However, unlike counting the number of recovered head capsules from parasitized hosts to determine host developmental stage at the time of parasitism, the stage of a host that was not parasitized could not be directly observed yet is required to estimate hazard rates. To circumvent this problem, we used previous data^45^ not reported in Holmes et al.^26^ to create probability distributions for each seed quality treatment that estimate which stage of development a host is in for each day of its development. We sampled age-specific stages from each seed quality probability distribution and assigned them to unparasitized host. We did this 100,000 times, each time estimating the hazard rates using Cox Proportional Hazard regression models from the *survival* package^46^ to create bootstrapped distributions of hazard coefficients across the three seed quality treatments for each stage of host development analyzed (e.g., L2, L3, L4, Pupal, and Adult). Due to only a single unsuccessful parasitism event, the first instar stage of host development was excluded from this analysis. The statistical model for each stage of host development (e.g., L2, L3, L4, Pupal, and Adult) was:

$${\text{Y}}\sim {\text{Q}}$$

where Y is a *survival* object of time (i.e., age of the host) and event (i.e., parasitism), and Q is the seed quality treatment (i.e., 90%, 95% and 100% black-eye pea flour).

Stage-specific null distributions of the hazard rates were estimated by assigning all individuals to a stage of development based on the same probability distributions used to assign unparasitized hosts a developmental stage and resampling all individuals without regard to their seed quality treatment 100,000 times. Hazard rates were estimated for each resampled dataset as described previously. The probability of observing differences in hosts’ mean daily risk of being parasitized among seed quality treatments was calculated for each stage of development from the stage-specific null distributions of Cox Proportional Hazard coefficients using the percentile method following Efron^47^.

Last, we noted 35 superparasitism events (i.e., more than one parasitoid egg was oviposited on a single host) that included 63 adult parasitoids and removed these individuals from parasitoid life history analyses.

## Results

**Figure 1:**
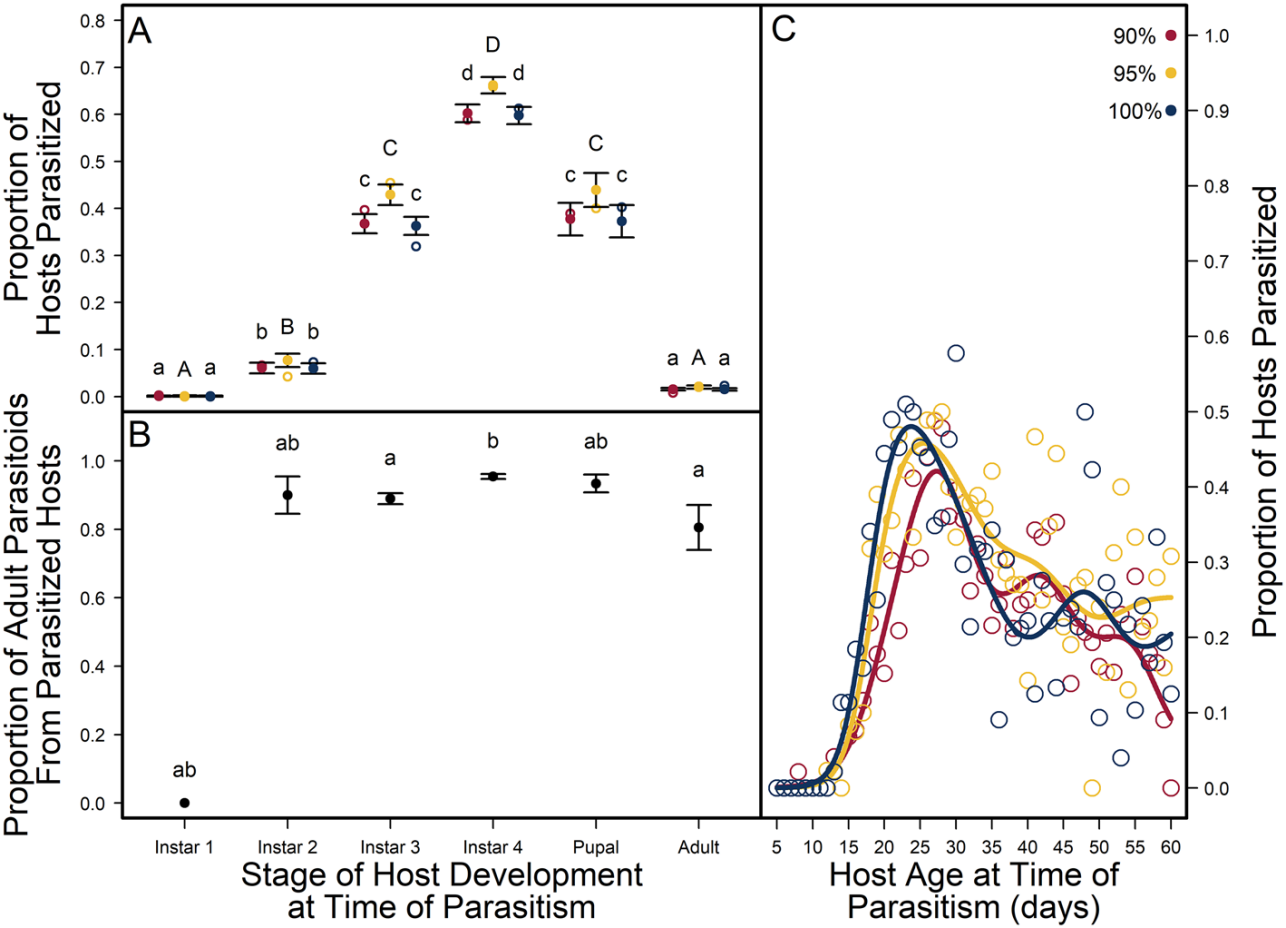
Parasitoid (*Anisopteromalus calandrae*) efficacy on stages of host (*Callosobruchus maculatus*) development (1st, 2nd, 3rd, and 4th larval instars, pupal, and adult) and host age across a food quality gradient of artificial seeds. (**A**) Observed proportion of hosts parasitized across stages of host development at the time of parasitoid attack for each seed quality treatment (90%, 95% and 100% black-eye pea flour) in red, yellow, and blue open circles, respectively, with fit proportions (± SE) in filled circles, respectively. (**B**) Proportion (± SE) of parasitized hosts that produced adult parasitoids across stages of host development at the time of parasitoid attack. Different letters across stages of host development indicate significant variation across host instars; lower and upper-case letters within the same stage of host development at the time of parasitism indicate a difference of means (± SE) across seed quality within host instars (p < 0.01). (**C**) Observed (circles) and predicted (lines) proportions of different aged hosts parasitized for each seed quality treatment (90%, 95% and 100% black-eye pea flour) in red, yellow, and blue, respectively.

The best model for predicting* A. calandrae* efficacy on *C. maculatus* included the independent effects of host seed quality and stage of host development (Table S1). Parasitoid efficacy generally increased with stage of host development (Fig. 1a), where fourth instar hosts were parasitized 23.69 ± 3.97% more than third instar hosts, but the proportion of parasitized hosts did not differ between third instar and pupal hosts. Generally, parasitism rates on first (n = 1) and second (n = 20) instar hosts and adult (n = 36) hosts were rare or low (Table S2). Parasitized first instar weevil hosts did not produce adult parasitoids (Fig. 1b) and only adult hosts that had not yet emerged from their seeds were parasitized.

Parasitoid efficacy also changed across the seed quality gradient that hosts consumed (Fig. 1a), where hosts consuming 95% quality seeds were parasitized 4.26 ± 2.55% more than hosts consuming 90% and 100% quality seeds, regardless of the stage hosts were parasitized in. We found no difference in the proportion of hosts parasitized across hosts consuming 90% and 100% quality seeds. Once a host was parasitized, host seed quality had no impact on successful development into an adult parasitoid, but stage of host development at the time of attack did (Fig. 1b; Table S3).

The relationship between the proportion of parasitized hosts and age of host at the time of parasitoid attack differed depending on the quality of seeds that hosts consumed (Fig. 1c; Table S4). Parasitoid efficacy on hosts consuming higher seed qualities (95% and 100%) showed similar trends of proportion of parasitized hosts for each host age at the time of parasitoid attack, while hosts consuming 90% quality seeds showed lower proportions of parasitism on hosts aged 15–25 days old at the time of parasitoid attack (Fig. 1c; Table S4).

**Figure 2:**
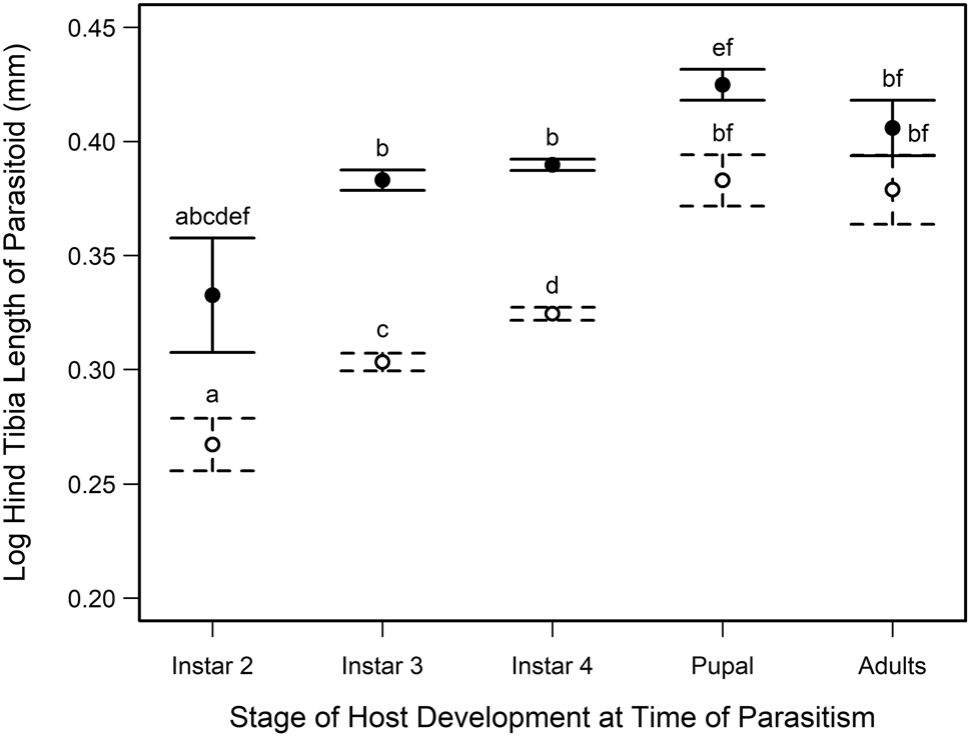
Log hind tibia length (mm) of female (solid circles) and male (open circles) parasitoid *Anisopteromalus calandrae*, developing on different stages of host, *Callosobruchus maculatus* development at the time of parasitoid attack. Different letters indicate significant variation in hind tibia length among stages of host development at time of parasitism and parasitoid sex (N = 1131). Note, hind tibia lengths for female parasitoids developing on 2nd instar hosts are not significantly different from parasitoids of either sex developing on any stage of host development (N = 4).

We found a strong correlation between parasitoid emergence mass and hind tibia length that differed by parasitoid sex (Fig. S2, Table S5). Both sexes have a steep positive relationship between emergence mass and hind tibia length (Fig. S2b), but females showed less variation in hind tibia length and more variation in emergence mass whereas males showed less variation in emergence mass and more variation in hind tibia length. Parasitoid hind tibia increased in size across host stages of development at the time of parasitism and females had longer hind tibias than males. But this sexual dimorphism in hind tibia length is greater among males and females that parasitized hosts in their second, third and fourth instar stages compared to hosts parasitized in their pupal and adult stages (Fig. 2; Table S6).

**Figure 3:**
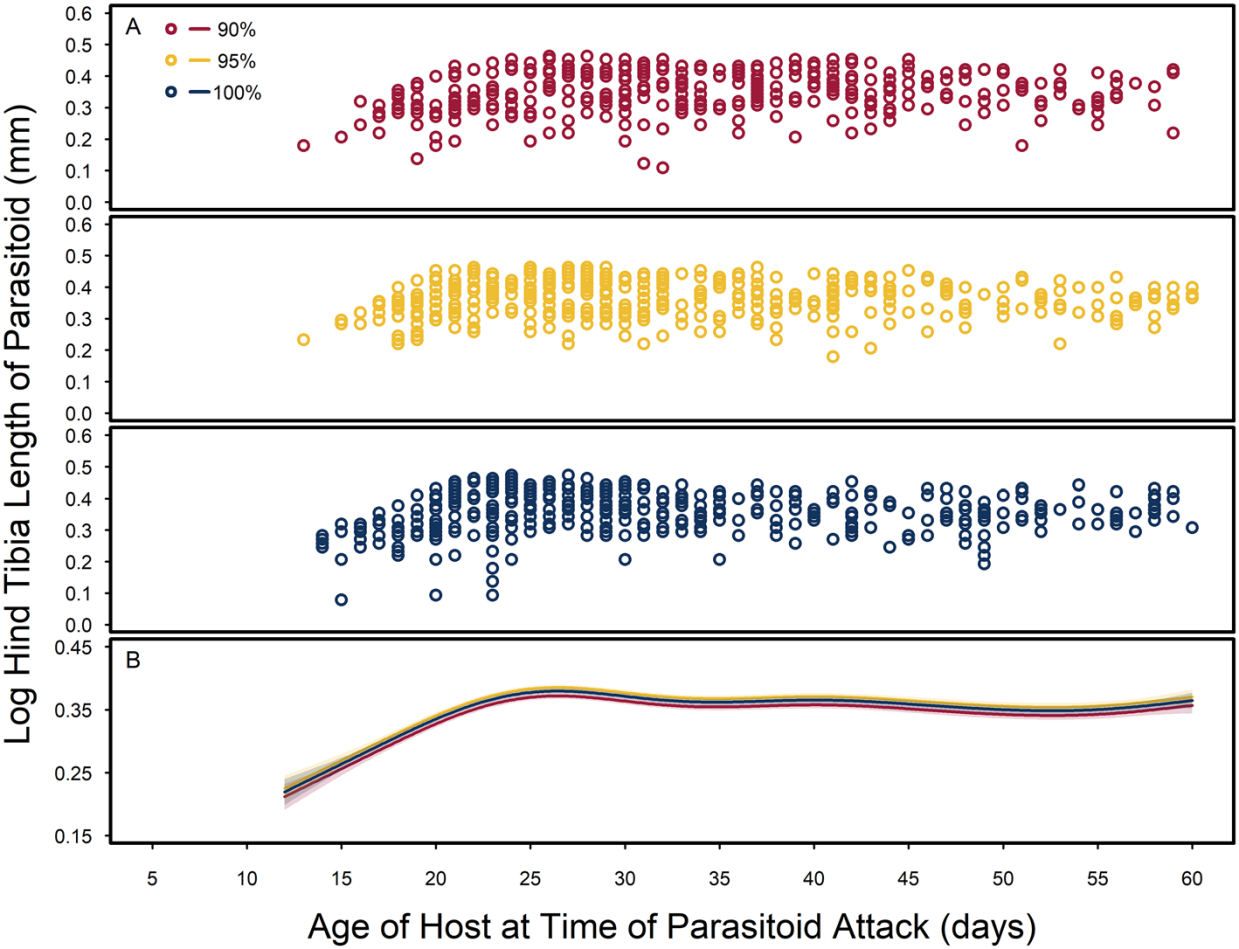
Log hind tibia length (mm) of parasitoid, *Anisopteromalus calandrae*, across host, *Callosobruchus maculatus*, age at the time of parasitoid attack for each seed quality treatment that hosts consumed. (**A**) Observed log hind tibia length of *A. calandrae* on parasitized hosts that consumed each type of seed quality (90%, 95% and 100% black-eye pea flour) in red, yellow, and blue, respectively. (**B**) Smoothing functions and 95% confidence intervals of log parasitoid hind tibia length (mm) across host age at the time of parasitism fit to each level of food quality treatment.

Host age at the time of parasitism also had an impact on parasitoid size (Fig. 3; Table S7). Host seed quality had a significant impact on parasitoid hind tibia length across host age; however, the effect size is quite small (Fig. 3). Like host stage-structure, parasitoid hind tibia length increased across parasitized host age-structure, but this relationship plateaus once hosts reach approximately 25 days old regardless of seed quality (Fig. 3; Table S7). We found slightly larger parasitoid hind tibia lengths on parasitoids that emerged from 23 to 27-day old hosts consuming the 95% and 100% quality seeds compared to hosts consuming the 90% quality seeds; however, we did not find an overall effect of host seed quality on parasitoid hind tibia length across host stage structure (Tables S6 and Tables S7).

**Figure 4:**
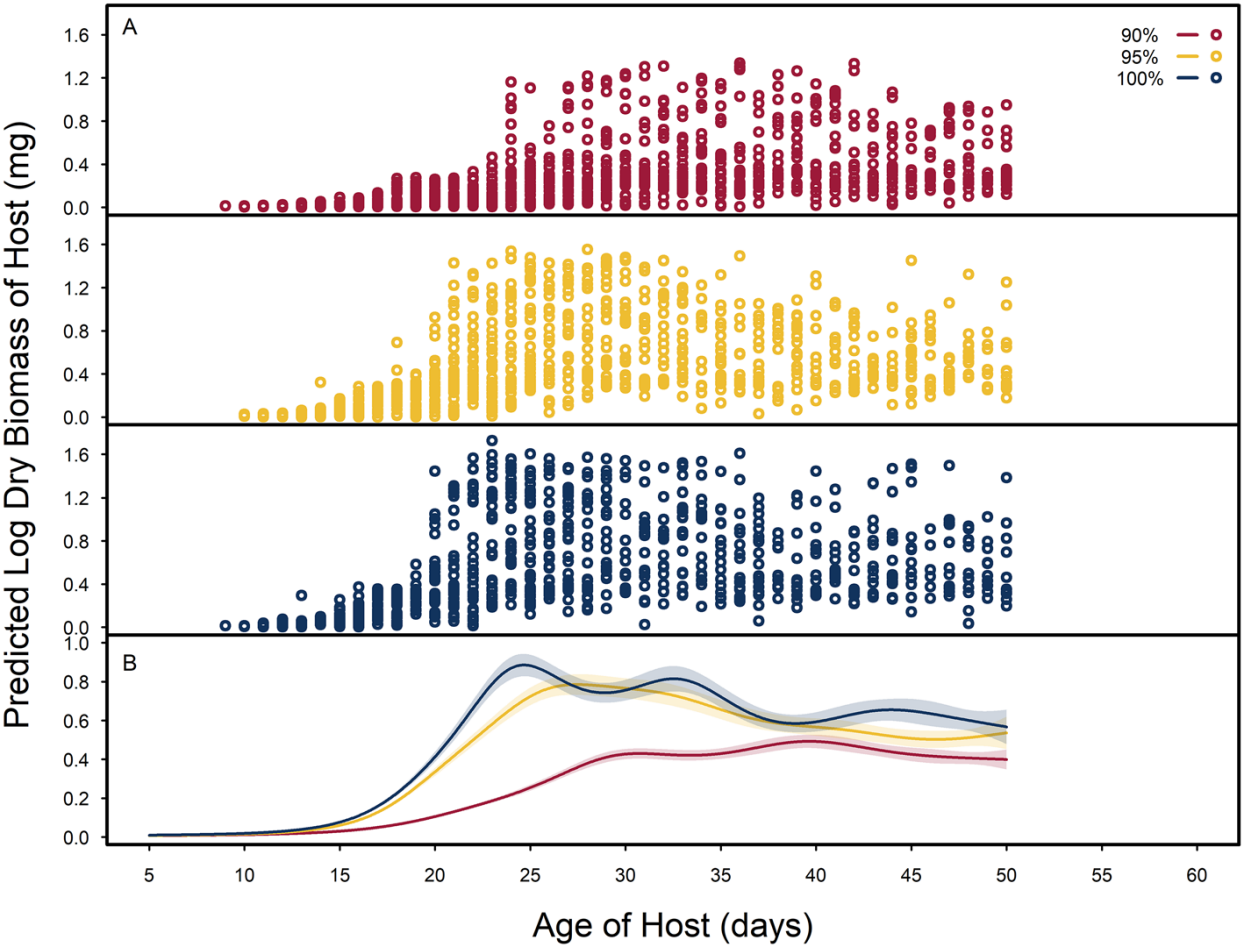
Log dry biomass (mg) of host, *Callosobruchus maculatus* that consumed a food quality gradient of artificial seeds across host age of development. Data are reported from a previously published dataset^45^. (**A**) Observed log dry biomass of hosts for each seed quality (90%, 95% and 100% black-eye pea flour) in red, yellow, and blue open circles, respectively. (**B**) Smoothing functions and 95% confidence intervals of host log dry biomass (mg) across host age fit to each level of food quality treatment.

The characterization of host growth showed the relationship between host dry biomass and age of host development can be predicted by the quality of seeds hosts consume (Fig. 4; Table S8). Smoothing functions of host age for host growth profiles that consumed higher seed qualities (95% and 100%) showed faster and larger growth trends than hosts consuming 90% quality seeds. Hosts consuming 100% quality seeds had slightly faster growth compared to hosts consuming 95% quality seeds. Otherwise, general growth trends were not different between these two seed qualities (Fig. 4).

Despite large impacts of seed quality on host growth (Fig. 4), parasitoid sex allocation differed across stages and ages of parasitized hosts at the time of parasitoid attack (Fig. S4; Tables S11 and S12) but differed little across host resource quality treatments (Fig. S4b). Generally, more female parasitoids emerged from hosts that were parasitized during later stages of host development (e.g., fourth instar hosts and pupal hosts) than males, and more male parasitoids emerged from hosts that were parasitized during younger stages of host development (e.g., second and third instar hosts) than females (Fig. S4a). This is corroborated by the proportion of female parasitoids allocated across host ages at the time of parasitism (Fig. S4c), where male parasitoids are primarily allocated to 20-day-old hosts or younger, while females are allocated to hosts aged 25–35 days. We found an even sex ratio allocation in hosts aged 20–25 days old, and over 35 days old; however, across host stage-structure, we found no evenness of sex allocation.

In addition to finding higher overall parasitism rates on hosts consuming 95% quality seeds, we found, a higher proportion of females allocated to hosts consuming 95% quality seeds, regardless of host stage at the time of parasitoid attack (Fig. S4b). However, when looking at the age-structure of the host, this effect of seed quality is only found for hosts parasitized 25 days old and older (Fig. S4c). Parasitoid development time also differed by sex. Host stage or age at the time of parasitism or the seed quality hosts consumed had no effect on parasitoid development time (Fig. S3, Tables S9 and S10). Female parasitoids took approximately one day longer than male parasitoids to develop (Fig. S3d). We note that several models including those that include the effect of seed quality and stage of host development at the time of parasitoid attack are less than 1 ΔAIC unit from the top model (Table S9); however, to be conservative, we present the findings from the top model. As a result, parasitoid sex is the only predictor of parasitoid development time.

**Figure 5:**
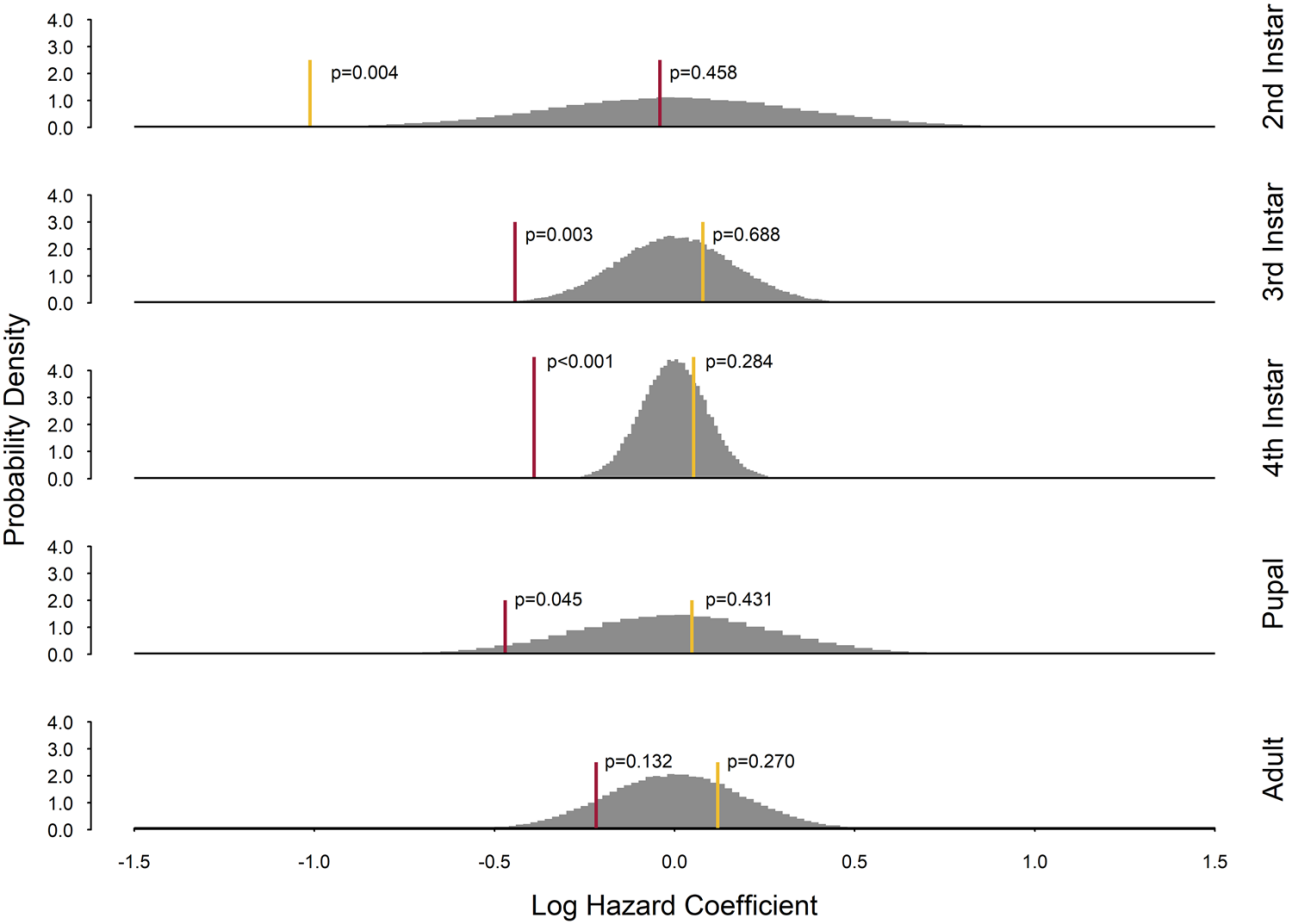
Null probability density distributions of *Callosobruchus maculatus* (host) daily risk of being parasitized by *Anisopteromalus calandrae* during each stage of host development (2nd instar, 3rd instar, 4th instar, pupal and adult) across an artificial seed quality gradient (90%, 95%, and 100% black-eye pea flour). Stage specific null distributions (in gray) of the log Cox Proportional Hazard rates (λ) estimated from 100,000 resampled parasitism events without regard to seed quality treatment. Yellow and red lines are the mean log hazard rates comparing 95% vs 100% and 90% vs 100% seed treatments, respectively. λ > 0 predicts an increased daily risk of parasitism, λ < 0 predicts a decreased daily risk of parasitism, and λ = 0 predicts no difference in daily risk of parasitism among seed quality treatments. First instar stages of host development were not included due to only one unsuccessful parasitism event observed in this experiment.

Null distributions (Fig. 5) of 100,000 bootstrapped Cox Proportional Hazard coefficients (λ) show mean daily risk of hosts consuming 90% or 95% seed qualities being parasitized compared to hosts consuming 100% seeds. We found hosts consuming 95% quality seeds have lower daily risk of being parasitized throughout their second instar stage compared to hosts consuming 100% quality seeds; however, we found no differences in daily risk of parasitism among hosts consuming 95% and 100% quality seeds in all other stages of host development (Fig. 5). Hosts consuming 90% quality seeds were estimated to have lower daily risk of parasitism in their third and fourth instar stages compared to hosts consuming 100% quality seeds, but we found no differences in daily risk of parasitism among host consuming 90% and 100% quality seeds in other stages of host development (Fig. 5).

## Discussion

Parasitoids are integral to ecosystem function and community assembly and their success depends on host life histories, which are largely impacted by food quality and its availability^19,48^. From our previous study^26^ and literature supporting bottom-up cascades of host resources in resource-host-parasitoid food webs^27^, we expected to find host resources indirectly impacting idiobiont parasitoid efficacy. Specifically, we expected hosts consuming lower seed qualities would be more susceptible to their idiobiont parasitoids due to longer periods of development (and thus, longer exposure to parasitoids). Instead, we found variations in host life histories across instar stages of host development are more important for parasitoid efficacy and life history than any variation within host instar in response to resource quality, despite large resource quality effects on host life histories^26^. While our results show some host food quality effects on host susceptibility to parasitoids, their effects on the scale of host age- and stage-structure reveal two interesting points.

Reporting on stage- and age-structure effects of host food quality on parasitoid efficacy reveals different patterns that may help explain discrepancies found in the literature of host food resources scaling through resource-host-parasitoid communities. Host susceptibility to idiobiont parasitoids is characterized by a window of time where hosts are of sufficient size to support parasitoid offspring development. The longer hosts take to develop is thought to increase their exposure to parasitoids and therefore their susceptibility to parasitoids^11,22,49–51^. Despite lower artificial seed qualities causing delays in host development within and across host stages of development^26^, we found parasitoid efficacy increased with stage of host development at the time of parasitoid attack, and any host food quality effects on parasitoid efficacy were small (Fig. 1a). The cross-sectional design of our study lends an opportunity to characterize host resource effects on host susceptibility to parasitoids and parasitoid life history under two different ecological lenses. Under a stage-structure lens, our results lend some support for this slow-growth-high-mortality hypothesis^52^, where we find slower developing hosts that consume 95% quality seeds^26^ are parasitized 5% more than faster developing hosts consuming 100% quality seeds. However, under an age-structure lens, we find the story is not as simple as slow-growth, high-mortality.

Under an age-structure lens, we find lower proportions of parasitized 15–28-day-old hosts consuming 90% seeds compared to similar aged hosts consuming 95% and 100% seeds. This is corroborated by a reduced daily risk of parasitism for third and fourth instar hosts consuming 90% quality seeds (Fig. 5), where 20–40% of these 15–28-day-old hosts are developing through their third and fourth instar stages^26^. However, their reduced daily risk to parasitism disappears by the pupal stage, suggesting hosts that consume lower quality seeds are simply parasitized later in development, but not necessarily more than hosts consuming higher quality seeds.

The mismatch of seed quality effects on parasitoid efficacy across host stage- and age- structure may be explained by seed quality effects on host life histories. For example, hosts consuming 90% quality seeds are 10–60% smaller than hosts consuming 95% and 100% quality seeds throughout the same ages of host development^26^. Additionally, hosts consuming 95% quality seeds are only parasitized more than hosts consuming 100% and 90% quality seeds when hosts are 30 days old or older (Fig. 1c). This increase in parasitoid efficacy on hosts 30 days old and older may be due to host life history differences in response to seed quality treatments. By age 30, many hosts consuming 100% quality seeds have already begun or completed pupation and metamorphosis^26^ and therefore may be less attractive to parasitoids. Moreover, hosts consuming the lowest quality seeds are greatly delayed in their development and suffer from higher rates of mortality^26^. Thus, higher rates of parasitism on hosts consuming 95% quality seeds may just be an artifact of the population’s demographic structure.

Contrary to the literature^22,23,51^, our results suggest longer host development in response to low food quality may only delay parasitism by idiobiont parasitoids; not reduce it or increase it. However, this appears to depend on the degree of food quality reduction because we also find even slower developing hosts consuming 90% quality seeds^26^ are not parasitized more than their faster developing counterparts. This implies seed quality effects on host size and development are not additive across a seed quality gradient^26^. For example, a 5% reduction in seed quality (e.g., 100% vs 95%) results in hosts that develop 1.4% slower and are 15% smaller^26^, whereas a 5% reduction in seed quality (e.g., 95% vs 90%) results in hosts that develop 22% slower but are 46% smaller^26^. Thus, longer host development time is not always equated to larger hosts and higher parasitism efficacies^11,51^, suggesting there is more to host susceptibility to idiobiont parasitoids than slower host development.

Most notably, we found little evidence for host resources cascading to impact parasitoid size and development. While artificial seed quality was included in the best supported statistical models for characterizing parasitoid life histories, its effect sizes on parasitoid life history traits (e.g., hind tibia length, development time, and sex allocation) are small, and arguably biologically insignificant. The marked reduction in host size (up to 60% smaller^26^) in hosts consuming 90% quality seeds appear to have very little impact on the parasitoids that consumed them (Figs. 3b and 4b), suggesting effects of host resources are markedly reduced in higher trophic levels as others have found^53,54^.

Additionally, we found *A. calandrae* could parasitize all ontogenetic stages of *C. maculatus,* including fully developed sclerotized adult beetles that had yet to leave their seed. While unsuccessful on first instar hosts, parasitized hosts of all other host stages were of sufficient size to support parasitoid offspring development regardless of host food quality or stage of host development at the time of parasitism, implying *A. calandrae* has a rather low critical threshold for host size. As a result, *A. calandrae* shows much promise as a biocontrol agent of *C. maculatus*, with more than 50% of host populations parasitized and the ability to parasitize all host developmental stages.

Our study did not consider parasitoid behaviour or choice and introducing choice may yield different results. For example, parasitoid learning strategies and search images of relative host qualities available in host populations affect parasitoid efficacy and life history (see^55^ for review;^56^ for an example). Thus, given the low number of parasitism events on first and second instar and adult hosts in our study (Table S2), these host stages may not be commonly parasitized in nature where parasitoids can survey host populations for quality and target more suitable hosts.

Overall, as previously reported^15–18^, we found that host quality has a large impact on parasitoid life histories. While host size is often used as a proxy for host quality^31,57^, we found the variation in host life histories across stages of host development has a larger impact on host susceptibility to parasitoids and parasitoid life histories than any variation within host stages of development in response to seed quality. For example, parasitism rates among host stages differed by 10–40%, depending on the stage of host development parasitized, but parasitism rates within host stages differed by only 1–4% across seed quality (Fig. 1). Similarly, parasitoid hind tibia length varied up to 45% across stages of parasitized hosts (Fig. 2) but varied less than 1% across a food quality gradient within parasitized host stages (Fig. 3). Further, our results support the general observation that female parasitoids take longer to develop than males (Fig. S3), are allocated to older, later stages of hosts (Fig. S4), and are larger in body size with increasing host stage and age (Figs. 2, 3^57^). Therefore, at least as far as idiobiont parasitoids are concerned, size matters, but only in the context of the variation in host life histories throughout their ontogeny.

Idiobiont parasitoids cueing into variation in host life histories across host instars over the variation in host life histories within host instars in response to host resources, may be related to how idiobiont parasitoids of concealed hosts find their hosts. Specifically, idiobiont parasitoids of concealed hosts are thought to use their antennae and other appendages to relay information on their host^14^ and it has been suggested that the pulse’s cavity size may be detected through vibrational sounding to perceive host size^14,58,59^. Hosts molt their head capsule between instars and grow a new larger one. With each increase in head capsule size, hosts can consume more resources. It may be that the 1–10% difference in head capsule size across a food quality gradient within host instars^26^ is not as differentiable to searching idiobiont parasitoids as the 42–45% difference in head capsule width across host instars^26^ through vibrational sounding.

What remains unclear is why some studies show host resources cascading to impact parasitoids, while others show the absence of host resources cascading (reviewed in Kagata and Ohgushi^27^ and Vidal and Murphy^29^). We suggest that reasons for this dichotomy may lie in species life history strategies and how host resources change host quality. For example, for a solitary idiobiont ectoparasitoid with a low host critical size threshold, like *A. calandrae*, any variation in host size in response to host resources may simply not matter because of the relative size of the host and the parasitoid. While we did not quantify the biomass of parasitized hosts remains recovered after parasitoid emergence, we almost always found ‘leftover’ host remains in successfully parasitized hosts regardless of food quality treatment, suggesting even hosts consuming the lowest seed quality were more than large enough for this parasitoid.

Alternatively, koinobiont parasitoids that allow their hosts to continue feeding while parasitoid larvae develop on them may be impacted more by host resources due to the nature of a living host whilst feeding and the continuous ingestion and/or sequestering of plant allelochemicals by hosts, which has been shown to impact parasitoids^60,61^. Changes in host quality in response to plant allelochemicals can include host size^62^ as well as host biochemistry (e.g., nutrition, physiology, and toxicity)^63,64^; however, our findings suggest that cascading effects of plant allelochemicals on parasitoids will more likely be due to host biochemistry than variation in host size. Future studies should carefully consider life histories and parasitoid strategy in host-parasitoid communities; data from such studies may provide insights on whether host resources cascade to impact their respective parasitoids when characterizing the relationship and dynamics of host-parasitoid communities.

## Supplementary Information

Supplementary Information.

## Data Availability

Data is available from the Dryad Digital Repository 10.5061/dryad.3ffbg79n3.
