# Supplementary material for: Strong effects of food quality on host life history do not scale to impact parasitoid efficacy or life history
Source: Sci Rep. 2023 Mar 2;13:3528. doi: 10.1038/s41598-023-30441-1 (PMC9981602; doi:10.1038/s41598-023-30441-1)
Supplement: Supplementary file 1 — Supplementary Information. [file 41598_2023_30441_MOESM1_ESM.pdf]

Supporting Information for L.A. Holmes, W.M. Nelson, and S.C. Lougheed

Confirming the mating status of each female parasitoid used in our characterization of resource quality effects on parasitoid efficacy and life history

The mating status of each female parasitoid used in this experiment was confirmed in a secondary assay. After each parasitoid was removed from its experimental host, it was placed in a petri dish with approximately 20 black-eye peas with 16-day-old stock bean weevil hosts inside for 24h. Each black-eye pea comprised at least one host larva, however, many contained several host larvae. After 24h, the female parasitoid was removed from the stock hosts, and black-eye peas were monitored for either weevil or parasitoid emergence. Once parasitoids emerged, we confirmed parasitoid mating status by the presence of female offspring. If a female offspring emerged, mating status was identified as successful because female offspring can only result from mated mothers. If no parasitoid offspring emerged from host larvae, mating status was identified as unsuccessful. Female parasitoids were also categorized as unmated if all parasitoid offspring from stock hosts were males, where more than twenty parasitoid offspring resulted. This data was used to index our parasitoid data such that only data from mated female parasitoids were included in sex allocation results.

Testing for differences in parasitoid efficacy and life history

All statistical analyses were done using the R software environment version 3.6.2 (R Software Program, 2020). We used generalized linear models to characterize parasitoid efficacy, emergence mass, tibia length, development time and sex ratio across stages of host development at the time of host parasitism and a food quality gradient of artificial seeds hosts consumed. Statistical significance was evaluated using model selection (Burnham & Anderson, 2002) with Akaike Information Criteria (AIC). Quasi Akaike Information Criteria (qAIC) was used where appropriate; their values calculated using the bbmle package (Bolker & R Development Core Team, 2017). The general linear hypothesis testing function glht in the Multcomp package (Hothorn, Bretz, & Westfall, 2008) was used for post-hoc analyses where applicable; p values were adjusted for Type I error using the Bonferroni method.

References

Bolker, B., & R Development Core Team (2017). Tools for general maximum likelihood estimation. Version 1.0.20.

Burnham, K. P. & Anderson, D. R. (2002). Model selection and inference: a practical information-theoretical approach (2nd Ed.). New York. Springer-Verlag.

Hothorn, T., Bretz, F., & Westfall, P. (2008). Simultaneous inference in general parametric models. Biometical Journal, 50, 346-363.

R Core Team. (2020). R: A language and environment for statistical computing. R Foundation for Statistical Computing, Vienna, Austria. URL https://www.R-project.org/

Table S1: Model selection for the effect of both artificial seed quality (Q) (90% black-eye pea:10% filler, 95% black-eye pea: 5% filler, and 100% black-eye pea:0% filler) consumed by hosts parasitized, and stage of host development at time of parasitism (S) (1st, 2nd, 3rd, 4th instar, pupal and adult) on the proportion of hosts parasitized, Y, fit to 6140 observations with 6617 null deviance.

Model	ΔqAIC	Df	Weight	Residual Deviance
Y ~ Q + S	0.0	8	0.752	4062.5
Y ~ Q * S	2.4	18	0.231	4088.9
Y ~ S	7.6	6	0.017	4078.4
Y ~ Q	2796.1	3	< 0.001	6604.9
Y ~ 1	2805.5	1	< 0.001	6617.0

Table S2: The number of parasitized hosts in each stage of host development (1st, 2nd, 3rd, 4th instar, pupal and adult) across each seed quality treatment (90% black-eye pea:10% filler, 95% black-eye pea: 5% filler, and 100% black-eye pea:0% filler).

Stage	90%	95%	100%
1st Instar	1	0	0
2nd Instar	14	4	11
3rd Instar	110	114	106
4th Instar	228	271	270
Pupal	25	31	28
Adult	6	15	14

Table S3: Model selection for the effect of both artificial seed quality (Q) (90% black-eye pea:10% filler, 95% black-eye pea: 5% filler, and 100% black-eye pea:0% filler) consumed by hosts parasitized, and stage of host development at time of parasitism (S) (2nd, 3rd, 4th instar, pupal and adult) on the proportion of adult parasitoids that emerged from parasitized hosts, Y, fit to 1410 observations with 716.81 null deviance.

Model	ΔAIC	Df	Weight	Residual Deviance
Y ~ S	0.0	6	0.674	697.9
Y ~ Q + S	1.5	8	0.322	699.4
Y ~ S * Q	10.4	16	0.004	708.3
Y ~ 1	20.9	1	< 0.001	718.8
Y ~ Q	22.4	3	< 0.001	720.3

Table S4: Model selection for the effect of artificial seed quality (Q) (90% black-eye pea:10% filler, 95% black-eye pea: 5% filler, and 100% black-eye pea:0% filler) consumed by hosts on the relationship of host age at the time of parasitoid attack (A) and proportion of parasitized hosts, (Y), fit to 6140 observations with 6617 null deviance. GAM indicates generalized additive models with a smoothing function, GLM indicates generalized linear models.

Model	ΔAIC	Df	Weight	Residual Deviance
GAM: Y ~ Q + s(A, by = Q)	0.00	24	0.99	5662.9
GAM: Y ~ s(A) + Q	8.9	11	0.012	5699.0
GAM: Y ~ s(A)	18.7	9	< 0.001	5712.8
GLM: Y ~ A * Q	799.0	6	< 0.001	6498.4
GLM: Y ~ A + Q	800.0	4	< 0.001	6503.4
GLM: Y ~ A	810.1	2	< 0.001	6517.6
GLM: Y ~ Q	899.4	3	< 0.001	6604.9
GLM: Y ~ 1	907.5	1	< 0.001	6617.0

Table S5: Model selection for the effect of parasitoid sex (G) (male or female) on the relationship of adult parasitoid hind tibia length (T) and emergence mass, (Y), fit to 375 observations with 3.17 null deviance.

Model	ΔAIC	Df	Weight	Residual Deviance
Y ~ T * G	0.00	5	1	0.5370
Y ~ T + G	16.25	4	< 0.001	0.5638
Y ~ T	36.93	3	< 0.001	0.5990
Y ~ G	487.74	3	< 0.001	1.9932
Y ~ 1	660.03	2	< 0.001	3.1728

Table S6: Model selection for the effect of artificial seed quality (Q) (90% black-eye pea:10% filler, 95% black-eye pea: 5% filler, and 100% black-eye pea:0% filler) consumed by hosts parasitized, stage of host development at time of parasitism (S) (2nd, 3rd, 4th instar, pupal and adult), and parasitoid sex (G) (male or female) on parasitoid hind tibia length, (Y), fit to 1131 observations with 4.62 null deviance.

Model	ΔAIC	Df	Weight	Residual Deviance
Y ~ G * S	0.00	11	0.495	2.82
Y ~ G * S + Q	1.71	13	0.210	2.81
Y ~ G * S + Q * S	2.91	21	0.115	2.78
Y ~ G * S + G * Q	4.66	15	0.048	2.84
Y ~ G + S	4.79	7	0.045	2.85

Table S7: Model selection for the effect of artificial seed quality (Q) (90% black-eye pea:10% filler, 95% black-eye pea: 5% filler, and 100% black-eye pea:0% filler) consumed by hosts on the relationship of host age at the time of parasitoid attack (A) and parasitoid hind tibia length, (Y), and parasitoid sex (G) (male or female), fit to 1131 observations with 4.7 null deviance. GAM indicates generalized additive models with a smoothing function, GLM indicates generalized linear models.

Model	ΔAIC	Df	Weight	Residual Deviance
GAM: Y ~ Q + G + s(A, by = Q) + s(A, by = G)	0.00	20	0.6107	2.8
GAM: Y ~ G + s(A, by = G)	1.2	14	0.3370	4.0
GLM: Y ~ $A^4 + A^3 + A^2 + A + A * Q * G$	6.6	16	0.0228	2.8
GAM: Y ~ G + s(A)	7.4	9	0.0153	2.9
GAM: Y ~ Q + G + s(A)	8.8	11	0.0074	4.0
GLM: Y ~ $A^3 + A^2 + A + A * Q * G$	9.0	15	0.0068	2.9
GLM: Y ~ $A^2 + A + A * Q * G$	43.4	14	< 0.001	4.2
GLM: Y ~ A * Q * G	63.4	13	< 0.001	3.0

Table S8: Model selection for the effect of artificial seed quality (Q) (90% black-eye pea:10% filler, 95% black-eye pea: 5% filler, and 100% black-eye pea:0% filler) consumed by hosts on the relationship of host age of development (A) and host dry biomass, (Y), fit to 2835 observations with 4009.5 null deviance. GAM indicates generalized additive models with a smoothing function, GLM indicates generalized linear models.

Model	ΔAIC	Df	Weight	Residual Deviance
GAM: Y ~ Q + s(A, by = Q)	0.00	31	1	1348.8
GAM: Y ~ s(A) + Q	382.0	13	< 0.001	1545.3
GAM: Y ~ s(A)	745.7	11	< 0.001	1739.8
GLM: Y ~ A * Q	2751.5	7	< 0.001	3255.7
GLM: Y ~ A + Q	2854.3	5	< 0.001	3361.6
GLM: Y ~ A	3044.1	3	< 0.001	3561.5
GLM: Y ~ Q	3281.3	4	< 0.001	3817.9
GLM: Y ~ 1	3444.3	2	< 0.001	4009.5

Table S9: Model selection for the effect of artificial seed quality (Q) (90% black-eye pea:10% filler, 95% black-eye pea: 5% filler, and 100% black-eye pea:0% filler) consumed by hosts parasitized, stage of host development at time of parasitism (S) (2nd, 3rd, 4th instar, pupal and adult), and parasitoid sex (male or female) on the development time of parasitoids that emerged from parasitized hosts, (Y), fit to 1197observations with 10.88 null deviance.

Model	ΔAIC	Df	Weight	Residual Deviance
Y ~ G	0.00	3	0.196	9.58
Y ~ G + Q	0.24	5	0.174	9.55
Y ~ G * Q	0.82	7	0.130	9.52
Y ~ G + S	0.84	7	0.129	9.52
Y ~ G + S + Q	1.07	9	0.115	9.49
Y ~ G * T + S	1.62	11	0.087	9.46

Table S10: Model selection for the effect of artificial seed quality (Q) (90% black-eye pea:10% filler, 95% black-eye pea: 5% filler, and 100% black-eye pea:0% filler) consumed by hosts on the relationship of host age at the time of parasitoid attack (A) and parasitoid development time, (Y) with parasitoid sex (G) (male or female) as a covariate, fit to 1197observations with 10.89 null deviance. GAM indicates generalized additive models with a smoothing function, GLM indicates generalized linear models.

Model	ΔAIC	Df	Weight	Residual Deviance
GAM: Y ~ G + s(A)	0.00	6	0.3409	9.4
GAM: Y ~ G + s(A, by = G)	0.3	10	0.2897	10.8
GAM: Y ~ G + Q + s(A)	1.3	8	0.1747	9.4
GAM: Y ~ G + Q + s(A, by = G)	1.8	12	0.1405	9.4
GAM: Y ~ G + Q + s(A, by = G) + s(A, by = Q)	5.7	14	0.0194	10.8
GLM: Y ~ A^2 + A + A * Q * G	6.3	14	0.0149	9.4
GLM: Y ~ A^3 + A^2 + A + A * Q * G	6.5	15	0.0131	10.9
GLM: Y ~ A^4 + A^3 + A^2 + A + A * Q * G	8.4	16	0.0051	9.4
GAM: Y ~ G + Q + s(A, by = Q)	10.6	13	0.0017	9.4
GLM: Y ~ G * Q * A	17.7	13	< 0.001	9.5
GAM: Y ~ s(A)	162.7	4	< 0.001	9.4
GAM: Y ~ Q + s(A)	164.5	6	< 0.001	9.4
GAM: Y ~ Q + s(A, by = Q)	168.7	8	< 0.001	9.4

Table S11: Model selection for the effect of artificial seed quality (Q) (90% black-eye pea:10% filler, 95% black-eye pea: 5% filler, and 100% black-eye pea:0% filler) consumed by hosts parasitized and stage of host development at time of parasitism (S) (2nd, 3rd, 4th instar, pupal and adult) on the sex ratio of parasitoids that emerged from parasitized hosts, (Y), fit to 1158 observations with 1599.8 null deviance.

Model	ΔAIC	Df	Weight	Residual Deviance
Y ~ S + Q	0.00	7	0.509	1547.3
Y ~ S	0.08	5	0.488	1551.4
Y ~ S * Q	10.21	15	0.003	1541.2
Y ~ Q	39.34	3	< 0.001	1594.7
Y ~ 1	40.41	1	< 0.001	1599.8

Table S12: Model selection for the effect of artificial seed quality (Q) (90% black-eye pea:10% filler, 95% black-eye pea: 5% filler, and 100% black-eye pea:0% filler) consumed by hosts parasitized and age of host development at time of parasitism (A) on the sex ratio of parasitoids that emerged from parasitized hosts, (Y), fit to 1158 observations with 1599.8 null deviance.

Model	ΔAIC	Df	Weight	Residual Deviance
GAM: Y ~ Q + s(A)	0.0	9	0.6291	1525.6
GAM: Y ~ s(A)	1.7	7	0.2696	1530.7
GLM: Y ~ $A^4 + A^3 + A^2 + A + A * Q$	3.8	9	0.0928	1536.3
GLM: Y ~ $A^3 + A^2 + A + A * Q$	9.1	8	0.0066	1594.7
GAM: Y ~ Q + s(A, by = Q)	11.5	17	0.0020	1599.8
GLM: Y ~ $A^2 + A + A * Q$	23.2	7	< 0.001	1593.9
GAM: Y ~ Q	51.8	3	< 0.001	1558.1
GAM: Y ~ 1	52.9	1	< 0.001	1542.1
GLM: Y ~ A * Q	57.0	6	< 0.001	1534.8

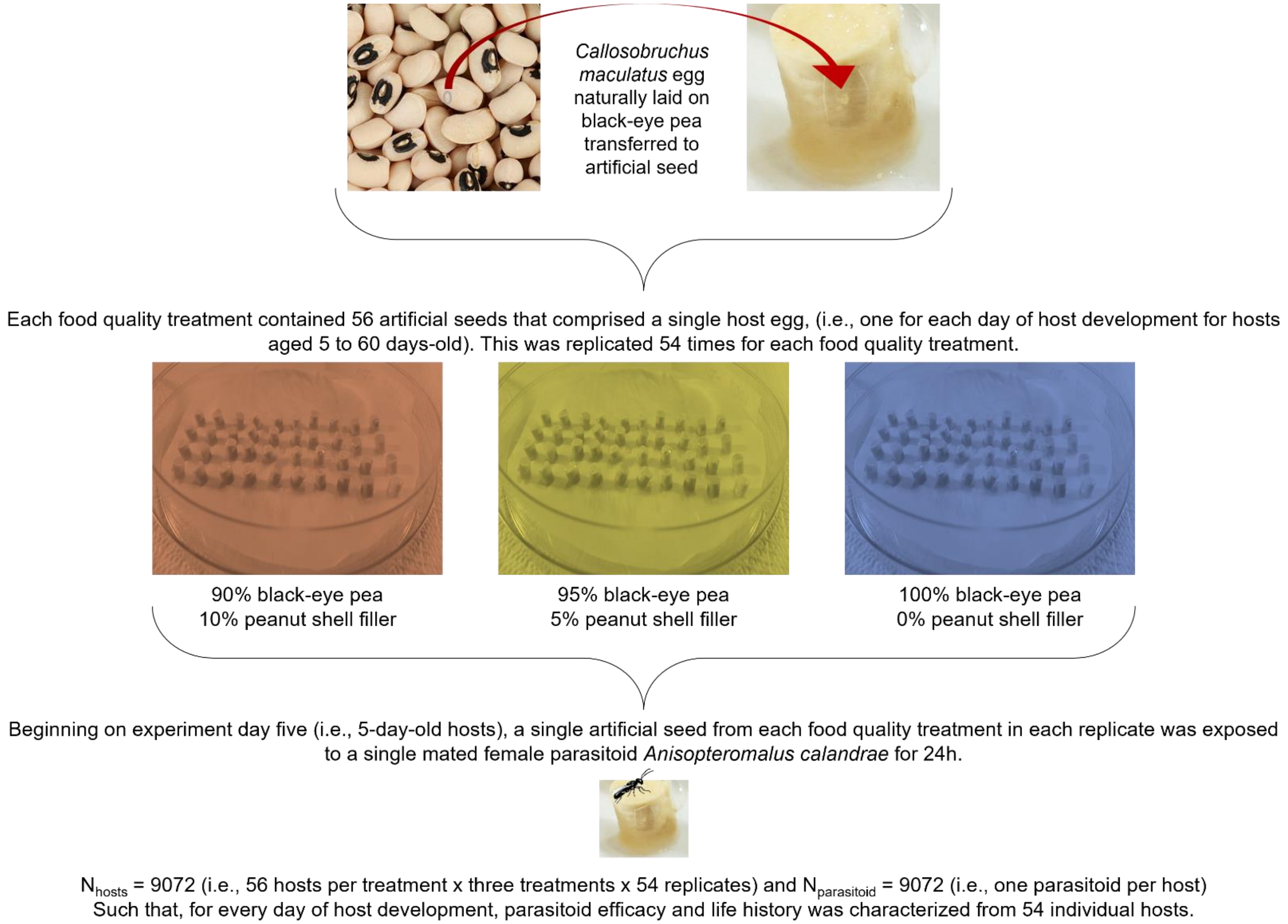

Figure S1 Visual abstract of experimental setup. In a cross-sectional experiment, *C. maculatus* hosts aged 5 to 60 days-old were exposed to their idiobiont ectoparasitoid *A. calandrae.* Due to the 'destructive' nature of sampling in this experiment, to capture host susceptibility to parasitoids and any scaling of host resources on parasitoid life history across the host's entire development, a sampling without replacement design was required. The cross-sectional design of this experiment enables data characterization on an age- and stage- structure scale with a resolution of 24h in host age-structure.

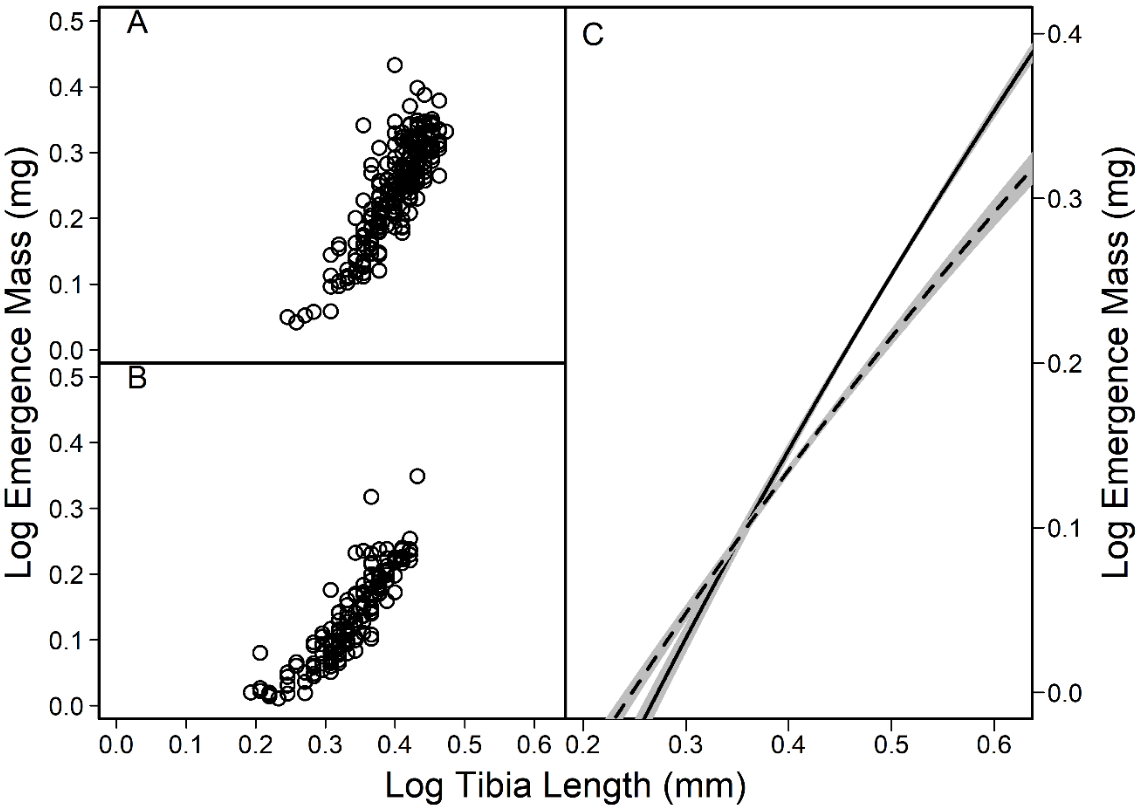

Figure S2 Log parasitoid (*Anisopteromalus calandrae*) emergence mass (mg) and hind tibia length (mm). A) Observed female emergence mass and hind tibia length. B) Observed male emergence mass and hind tibia length. C) Model fitting the relationship of parasitoid emergence mass and hind tibia length as predicted by parasitoid sex (Solid line is females and dashed line is males).

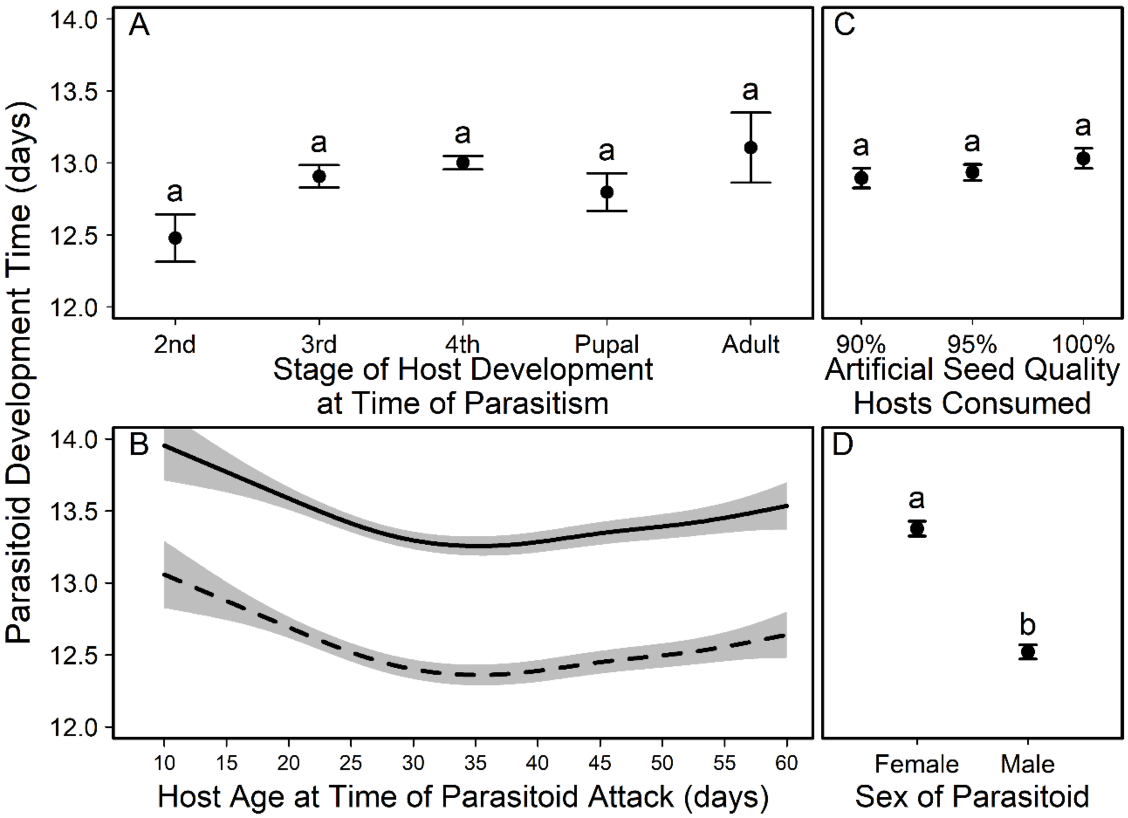

Figure S3 Parasitoid (*Anisopteromalus calandrae*) development time (days) on stages of host (*Callosobruchus maculatus*) development (2nd, 3rd, and 4th larval instars, pupal, and adult) and host ages consuming a food quality gradient of artificial seeds (90%, 95% and 100% black-eye pea flour). A) Mean (±SE) number of days parasitoids took to develop on different stages of host development at the time of parasitoid attack. B) Top model fit of parasitoid development time and age of parasitized host as predicted by parasitoid sex (solid line is females and dashed line is males). C) Mean (±SE) number of days parasitoids took to develop on hosts consuming different seed qualities. D) Mean (±SE) number of days male and female parasitoids took to develop on hosts regardless of host stage or seed quality consumed by parasitized hosts. Means (±SE) with different letters within a plot are significantly different ($p < 0.05$).

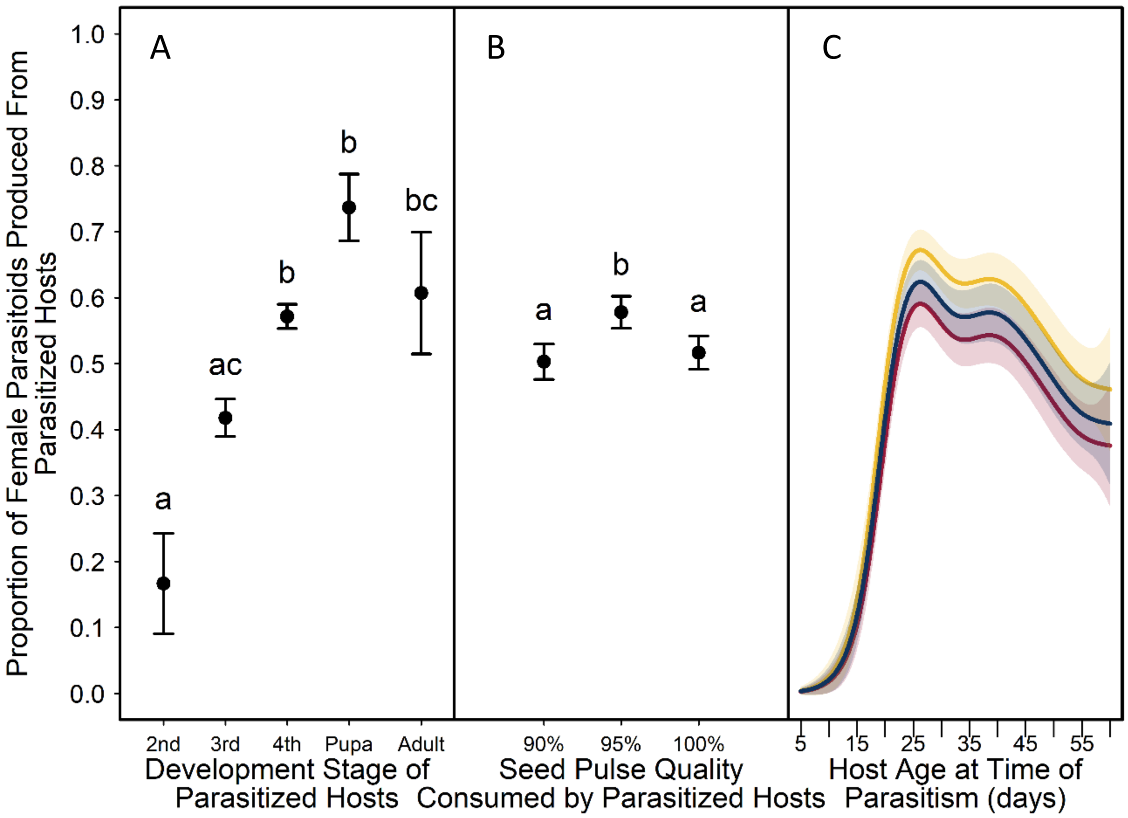

Figure S4 The proportion of female parasitoids (*Anisopteromalus calandrae*) that emerged from *Callosobruchus maculatus* hosts: A) parasitized at different host stages (2nd, $3^{rd,}$ and 4th instar, pupal and adult) and B) consuming different seed qualities (90%, 95% and 100% black-eye pea flour). Means (±SE) with different letters within a plot are significantly different ($p < 0.05$). C) Smoothing functions and 95% confidence intervals of proportions of female parasitoids emerging from different aged hosts fit to each level of seed quality in red, yellow and blue, respectively.
